# Microparasites and Placental Invasiveness in Eutherian Mammals

**DOI:** 10.1371/journal.pone.0132563

**Published:** 2015-07-13

**Authors:** Isabella Capellini, Charles L. Nunn, Robert A. Barton

**Affiliations:** 1 School of Biological, Biomedical and Environmental Sciences, University of Hull, Hull, United Kingdom; 2 Department of Evolutionary Anthropology & Duke Global Health Institute Biological Sciences, Duke University, Durham NC, United States of America; 3 Department of Anthropology, Durham University, Durham, United Kingdom; CSIRO, AUSTRALIA

## Abstract

Placental invasiveness—the number of maternal tissue layers separating fetal tissues from maternal blood—is variable across mammalian species. Although this diversity is likely to be functionally important, variation in placental invasiveness remains unexplained. Here we test the hypothesis that increased risk of transplacental transmission of pathogens from the mother to the fetus promotes the evolution of non-invasive placentation, the most likely derived condition in eutherian mammals. Specifically, we predict that non-invasive placentation is associated with increased microparasite species richness relative to more invasive placental types, based on the assumption that higher numbers of microparasites in a population reflects greater risk of transplacental transmission to fetuses. As predicted, higher bacteria species richness is associated with non-invasive placentation. Protozoa species richness, however, shows the opposite pattern. Because invasive placentae facilitate the transfer of maternal antibodies to the fetus, we propose that the ancestral condition of invasive placentation is retained under selection for protection of newborns from higher risk of postnatal protozoan infection. Hence, our findings suggest that a tradeoff exists between protection against bacterial infection prenatally and protozoan infection postnatally. Future studies are needed to investigate how maternal prevalence of infection and the relative pre- versus postnatal risk of fetal infection by different microparasite groups vary among mammalian hosts in relation to placental invasiveness.

## Introduction

The placenta is intrinsic to mammalian reproduction and fetal development; it nourishes the developing fetus by transferring nutrients and oxygen from the mother’s bloodstream to the fetus, eliminates the waste products of fetal metabolism, and protects the embryo within its fluid filled cavity [1,2]. Despite undertaking these same functions in all mammals, the placenta exhibits great morphological diversity among species, but the selective pressures that are responsible for the evolution of placental diversity remain poorly understood [2–4]. One placental characteristic that varies between species is placental invasiveness, i.e. the nature of the contact between maternal and fetal tissues [1–4].

Placental invasiveness is defined by the number of maternal tissue layers at the areas of exchange separating the maternal blood from the external tissues of fetal origin (the ‘trophoblast’) [2–4]. In non-invasive epithelichorial placentae, such as those of artiodactyls and lemurs, the maternal tissue layers (uterine epithelium, connective tissues and endothelium of the blood vessels) remain intact and opposed to fetally derived tissues, thus maintaining a physical barrier between trophoblast and maternal blood. In intermediately invasive endotheliochorial placentae, which are found in most carnivores, the trophoblast erodes the maternal epithelium and is in contact with the endothelial walls of the maternal blood vessels. Lastly, in highly invasive hemochorial placentae, such as those of anthropoid primates, the endothelium of maternal blood vessels is also eroded and the trophoblast tissues are bathed directly in maternal blood [2–4].

Increased placental invasiveness was long believed to facilitate fetal nutrition because the direct contact with maternal blood should allow easier fetal access to maternal resources and ultimately lead to enhanced fetal growth rates [5–7]. However, Capellini et al. [8] recently showed that the evolutionary changes in placental invasiveness across the phylogenetic tree of mammals are unrelated to fetal brain and body growth rates. Instead, they found that growth rates depend on the dimensions of the surface area of exchange (‘interdigitation’ [9]), irrespective of placental invasiveness. Moreover, the non-invasive epitheliochorial placentation appears to be a derived trait that evolved several times independently in eutherian mammals from more invasive placental types (hemochorial or endotheliochorial) [10–12]. Thus, it is the evolution of less invasive placentae from more invasive placentae that requires explanation. Here we test the hypothesis that parasite pressure provides such an explanation.

Transmission of infectious organisms across the placenta is well known [1,13–15]. Fetal and placental infection compromise fetal health and growth pre- and postnatally, and may also cause abortion or stillbirth [1,13–15]. Thus, placental and fetal infection are detrimental to maternal and offspring fitness. Microparasites that can infect and cross the placenta include viruses (e.g. *Cytomegalovirus*, HIV), protozoa (e.g. *Toxoplasma*, *Trypanosoma*, *Leishmania*, *Plasmodium*), and bacteria (e.g. *Treponema*, *Brucella*, *Listeria*) [14–17]. Among macroparasites, some helminths have also been observed to cross the placental barrier (e.g. *Toxocora*, [1,13], and *Trichinella* [18]). Loke [13] suggests that the direct contact with maternal blood in hemochorial placentae might facilitate placental infection and transmission of organisms from the placenta to the fetus.

Non-invasive placentation may help oppose transplacental infection by allowing a more limited local modulation of the maternal immune system during gestation. The implantation of the trophoblast, in fact, poses a challenge for intrauterine fetal development because the recognition of the trophoblast as non-self by the maternal immune system could induce rejection [19]. The implantation of the trophoblast thus requires adaptations in both the mother and the trophoblast. While this problem has been often compared to organ transplantation, in recent years it has become apparent that the trophoblast adopts similar strategies to those observed in pathogens or commensal bacteria, as it diverts or evades the immune system of the mother [19–21]. Specifically, irrespective of placental invasiveness, the trophoblast expresses the least polymorphic MHC genes, hence reducing the opportunity of detection by the maternal immune system [19,21–27].

During pregnancy the maternal immune system is also modulated locally to reduce the risks of rejection of the fetus [2,19,28]. Given that invasive placentation is characterized by direct contact between fetal tissues and maternal blood (and thus maternal immune cells), changes in the profile of maternal immunity at the local level are suggested to be more important with this mode of placentation [2]. Natural killer cells represent the first line of defence against pathogens and are present in the uterus in species with invasive hemochorial placentation [19]. Uterine natural killer cells, however, have poor defence ability and their role is instead to help remodel the maternal vasculature during implantation. They may also modulate the activity of immune cells within the decidua, including T cells and decidual macrophages, thus contributing to tolerance of the trophoblast [20,22,26,28]. In species with non-invasive epitheliochorial placentation, natural killer cells are generally not present at the placentation site. In a few species where they are present (e.g. in the cow), they appear to have natural cytotoxicity, and as a result the local population of T cells (e.g. αβ-TCR^+^, CD8^+^ and CD4^+^ T cells) is maintained or activated [20,27]. Furthermore, αδ-T cells in the uterus of pregnant ruminants, i.e. species with epitheliochorial non-invasive placentation, appear to have important antimicrobial properties [20]. Collectively, these findings suggest that non-invasive placentae might have evolved in response to high parasite pressure to maintain a barrier to infectious organisms by limiting immune system modulation at the implantation site during pregnancy [13,29].

While numerous studies have investigated the pathology of the placenta and fetus, only two have considered whether mammalian diversity in placental invasiveness varies in relation to the risk of placental and fetal infection. In a review on transplacental transmission of parasites, Loke [13] found evidence that *Toxoplasma* can infect a fetus more easily and frequently in species with hemochorial placentation relative to those with less invasive placentae. Moreover, Webster and Kapel [18] tested the hypothesis that the diversity in placental invasiveness affects fetal infection rates experimentally. They found that the likelihood of *Trichinella* crossing the placental barrier and infecting the fetus is higher in guinea pigs (*Cavia porcellus*) and mice, both with hemochorial placentation, than in mammals with less invasiveness placentae (specifically pigs with epitheliochorial placentae; and foxes, *Vulpes vulpes*, and ferrets, *Mustela putorius*, with endotheliochorial placentation).

Whilst these observations are consistent with the hypothesis that cross-placental parasite transmission is influenced by placental morphology, they do not rule out alternative explanations. Here we apply broader comparisons and phylogenetic statistical analysis to test more rigorously for such an association, using data on microparasite species richness and placentation in eutherian mammals. We predict that epitheliochorial (non-invasive) placentation is associated with greater microparasite species richness relative to hemochorial (highly invasive) placentation. The assumptions underlying our test are that (i) high presence of many potentially transmissible organisms in adults, especially females, favour placental adaptations to reduce transmission to more susceptible offspring, and (ii) the presence of these placental adaptations does not influence the observed levels of parasitism in the adult populations (otherwise, the selective pressure would be reduced). This latter assumption is justified because infection of fetuses may result in their death but less frequently maternal death [1,2,13], thus having little influence on disease prevalence and dynamics in populations.

## Methods

Here we carry out a phylogenetic comparative study on the correlated evolution between placental morphology and parasite richness across a sample of 138 mammalian species, using up to date phylogenetic comparative approaches, and specifically phylogenetic generalized least squares models that account for species’ shared ancestry (see below). We extract data on placental invasiveness at delivery from the literature (references in Appendix A in S1 File), following the protocol we have previously developed (described in Capellini et al. [8]). Because the taxonomy of some species has changed over time, we update any species’ names in the original studies that have changed to the currently accepted taxonomy by Wilson and Reeder [30]. This allows us to match the species’ names in the original source to those currently accepted, and so match the species’ data on placental morphology and parasite richness to the phylogeny of mammals that must be incorporated into comparative analysis across species (see below).

We define parasite species richness as the number of parasite species documented in a host species. If a parasite species has ever been documented in the host, it is counted towards richness for that host. Parasite richness does not include data on frequency and prevalence of infection; nor does it include evidence of disease, as such detailed data are not available for large sample of species in the wild. Parasite species richness is associated with rates of evolution in MHC diversity [31] and is considered a good proxy for parasite pressure [32,33]. Here we focus on parasite groups that are most frequently reported to infect and cross the placental barrier, i.e. viruses, protozoa and bacteria [1,13,15], hereafter called microparasites. Species richness for these microparasites is available in the open source *Global Mammal Parasite Database*, where information on host-parasite occurrence is reported for individuals of different mammalian hosts postnatally, with most records being from adults [34]. Following previous studies (e.g. [31,35]), we include citation counts of the host species in all our models to control for differences among mammalian species in research effort that affect estimates of parasite species richness. Our dataset includes 138 species of Primates, Artiodactyla, Perissodactyla and Carnivora (see S1 Data).

Species share phenotypic characteristics through their common ancestry [36,37]; for example, artiodactyls and cetaceans all have inherited non-invasive epitheliochorial placentation from their common ancestor [10–12]. The degree of similarity between species due to shared ancestry (‘phylogenetic signal’; [38]) depends on the species’ common evolutionary time, so that more closely related species resemble one another more than distantly related species [36,37]. This similarity among species due to shared ancestry creates a problem of ‘phylogenetic pseudoreplication’ when using standard statistical methods, as species’ phenotypic trait values are not independent data points but in part reflect shared evolution. Phylogenetic pseudoreplication therefore leads to violation of assumptions of statistical independence in analyses of data from different species, which often leads to incorrect conclusions about statistical significance [36,37,39,40]. It is therefore essential to account for species’ shared evolutionary history in statistical analyses across species. Phylogenetic comparative methods incorporate the evolutionary history of the studied species into the statistical models, as the topology and branch lengths of phylogenetic trees reflect the time of shared evolution between species, and allow the user to quantify and account for the phylogenetic signal in the data [36,37,39–44]. By contrast, analysis that include taxonomy instead of phylogeny do not adequately account for species’ shared ancestry as (i) they are based on arbitrarily defined taxonomic ranks; (ii) treat every taxonomic rank as equivalent; and thus (iii) ignore that similarity between species is a function of common evolutionary time [36, 37, 39, 41].

Here we employ up to date phylogenetic comparative methods that include the phylogeny of species in the statistical models. Specifically we use phylogenetic generalised least squares (PGLS) models, with maximum likelihood (ML) estimation of model parameters, to account for the importance of species’ shared ancestry [42–44]. We use the package *caper* in R [45] to run PGLS models and a mammal supertree with updated branch lengths [46] widely used in comparative studies. PGLS models are a very flexible approach as they can estimate the strength of the phylogenetic signal in the data with the λ parameter [42–44]. The λ parameter varies between 0 (species’ phenotypic traits are independent of species phylogenetic relationships) and 1 (the phenotypic similarity between species is proportional to the time of shared evolution under Brownian motion model of evolution [42–44]). In a statistical PGLS model, λ is estimated on the model residuals and accounts for one degree of freedom [42–44,47]. The significance of each predictor in PGLS models is assessed through t-statistics, the degrees of freedom (df) and associated p-value for its parameter estimate *β* [42,45]. We set the α level of significance for all analyses to 0.05.

We use PGLS models with citation count for the species as a covariate and microparasite species richness as the dependent variable. Placentation is coded with dummy variables [48] as described in Capellini et al. [8], setting epitheliochorial placentation as the reference level and testing *post-hoc* whether endotheliochorial and hemochorial placentation differ from one another. Log-transformation of microparasite species richness and citation count produce residuals that meet the assumptions of statistical models (Appendix B in S1 File). Because the number of microparasite species described in a host should plateau as sampling effort accumulates, we test whether a quadratic term for citation counts improves the fit to the data. The goodness of fit of alternative nested models, as quantified by their log-likelihood (Lh), is compared using likelihood ratio (LR) tests with df equal to the number of estimated parameters that differ between models [48]. In all models for bacteria and protozoa species richness, a polynomial model with a quadratic term for citation count significantly improves the fit to the data relative to a linear model (Table 1). Thus, we present models with a quadratic term when estimating the relationship between placentation and species richness of these microparasites. Finally, we check whether our results are influenced by the presence of outliers, i.e. residual model values outside the normal range [48]. Following standard practice, we define an outlier as a residual value grater than 3SD from the model fit line, and we reevaluate the model after outliers are excluded [45, 48]. Where outliers are present, we present models prior and after species that produce outliers in a model are excluded.

**Table 1:** Correlated evolution of species richness between the different microparasite groups.

Microparasite	Protozoa		Bacteria		Virus	
Predictors	t_132_	p	t_132_	p	t_133_	p
Citation count	-1.5	0.144	-2.5	0.015	3.2	0.002
(Citation count)^2^	2.3	0.026	2.8	0.005	-	-
Bacteria	2.4	0.016	-	-	6.2	<0.001
Virus	3.1	0.002	6.4	<0.001	-	-
Protozoa	-	-	2.6	0.010	2.8	0.006
**Model summary**						
Lh	-1.1		-11.1		-18.6	
ML λ	0.36		0.47		0.47	
R^2^	0.47		0.55		0.56	

Protozoa, bacteria and virus species richness are all positively associated with one another, after controlling for citation count (Table 1). Ignoring these associations might lead to spurious results when investigating the coevolution between a single microparasite group and placentation: an apparent significant association might reflect the association between the species richness of a second microparasite group and placentation when the species richness of microparasite groups covary. Therefore we include species richness of the other microparasites and citation counts in models that test the association of each microparasite group with placental invasiveness. However, models not controlling for species richness of other parasite groups give qualitatively similar results (Table A in S1 File).

For virus species richness, a PGLS polynomial model does not improve the fit to the data relative to a linear model for citation count [without (citation count)^2^: Lh = -18.6; with (citation count)^2^: Lh = -17.6; LR_1_ = 2.0, p = 0.157]. For each predictor in the model we report the t-value with degrees of freedom (t_df_) and p-value; for each model we report the model log-likelihood (Lh), the estimated value of the phylogenetic signal in the model residuals as quantified by λ (ML λ), and the amount of variance in microparasite species richness explained by the model (R^2^).

## Results

**Fig 1:**
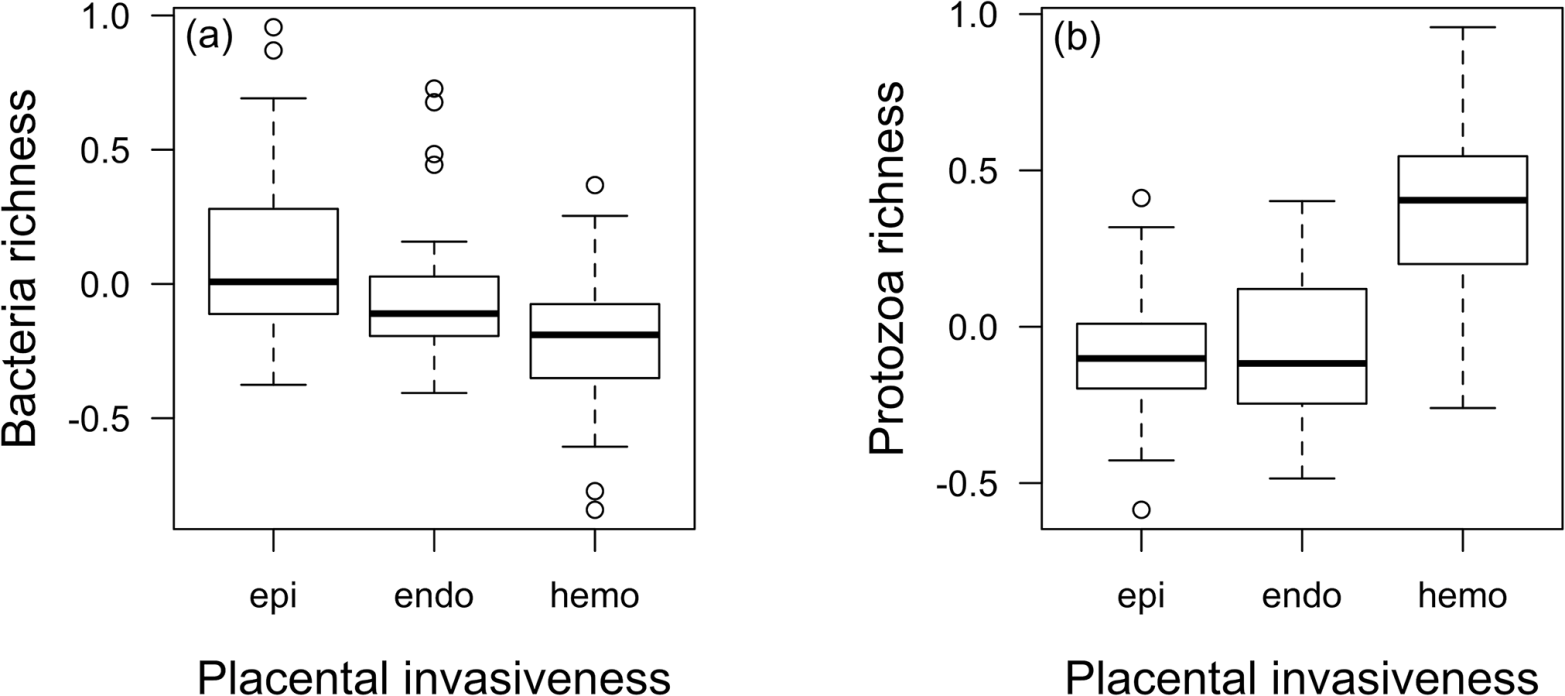
Placental invasiveness and microparasite species richness. Placental invasiveness and (a) Bacteria and (b) protozoa species richness, after accounting for citation count and species richness of the other microparasite groups (epi = epitheliochorial; endo = endotheliochorial, hemo = hemochorial).

As predicted, bacteria richness is higher in epitheliochorial relative to hemochorial placentation, while the intermediately invasive endotheliochorial placentation does not differ from both hemochorial and epitheliochorial placentation, after citation counts and other microparasite species richnesses are accounted for (Fig 1A, Table B in S1 File). The primate *Galagoides demidoff* is an outlier in this analysis, its residual being more than 3SD from the model line of best fit (outlier as defined in Methods based on references [45, 48]). After removing this species from the dataset, endotheliochorial placentation is significantly different from both epitheliochorial and hemochorial placentation (Table 2). Placental invasiveness, however, explains only an additional 3% of variance in bacteria species richness relative to a model without it, and the LR test indicates that a model with invasiveness does not significantly increase the fit to the data (LR_2_ = 3.2, p = 0.202).

**Table 2:** Full models of microparasite species richness and placentation while controlling for other microparasite groups.

Microparasite	Bacteria		Protozoa		Virus	
Predictors	t_129_	p	t_130_	p	t_131_	p
Citation count	-2.1	0.036	-1.2	0.227	3.1	0.002
(Citation count)^2^	2.7	0.008	2.0	0.048	-	-
Bacteria	-	-	2.5	0.013	6.1	<0.001
Virus	5.5	<0.001	3.0	0.003	-	-
Protozoa	2.6	0.010	-	-	2.7	0.007
Endotheliochorial	-2.2	0.028	0.2	0.866	0.6	0.528
Hemochorial	-5.6	<0.001	8.0	<0.001	0.1	0.882
***Post-hoc* testing**						
Epitheliochorial	5.6	<0.001	-8.0	<0.001	-0.1	0.882
Endotheliochorial	2.8	0.005	-6.7	<0.001	0.4	0.673
**Model summary**						
Lh	-9.8		5.1		-18.4	
ML λ	0.0		0.0		0.47	
R^2^	0.59		0.58		0.56	

Placentation is dummy coded (following [8], see Methods). Epitheliochorial placentation is used as the reference level; in *post-hoc* testing hemochorial placentation is set as the reference level. Results for bacteria species richness are reported for the analysis without *Galagoides demidoff* that is an outlier (see Results); results with *G*. *demidoff* are reported in Table B in S1 File. For each predictor in a model we report the t-value with degrees of freedom (t_df_) and p-value; for each model we report the model log-likelihood (Lh), the estimated value of the phylogenetic signal in the model residuals as quantified by λ (ML λ), and the amount of variance in microparasite species richness explained by the model (R^2^).

Contrary to predictions, protozoa species richness is significantly higher with hemochorial placentation than with less invasive endotheliochorial and epitheliochorial placentae, which do not differ from one another (Table 2, Fig 1B). A simpler model with placentation classified as hemochorial or not (i.e. lumping the less invasive endotheliochorial and epitheliochorial placentae together), explains an additional 11% of variance in protozoa species richness relative to a model without placentation, and significantly improves the fit to the data (LR_1_ = 12.4, p<0.001). Finally, virus species richness is not significantly related to placentation and placental invasiveness does not improve the fit to the data (LR_2_ = 0.4, p = 0.819).

## Discussion

We find mixed support for the hypothesis that higher risk of transplacental transmission of infectious organisms from mother to fetus promotes the evolution of non-invasive placentation [13]. We wish to emphasize that we made the *a priori* prediction of a link between non-invasive placentation and higher microparasite species richness in a host species. While non-invasive placentation is associated with greater bacteria species richness as predicted, we find an opposite pattern for protozoa species richness and placental invasiveness, and virus species richness is not significantly related to placentation. Overall, our results highlight the intriguing possibility that different microparasites might affect the evolution of placental morphology in different ways.

Consistent with Loke’s [13] hypothesis, bacteria species richness is higher in species with non-invasive epitheliochorial placentation, intermediate with intermediately invasive endotheliochorial placentation, and lowest in mammals with the most invasive hemochorial placentae. The association between placental invasiveness and bacteria species richness, however, explains a small proportion of variance in the data. We suggest that this low amount of variance explained might relate to two factors: the transfer of commensal bacteria and the presence of additional defense mechanisms in hemochorial placentae. Contrary to the common belief that placental and neonatal bacterial infections mostly originate from the maternal genital tract, a recent study shows that the microbiome of the human placenta originates from the microbiome of the maternal mouth, is low in abundance but taxonomically very diverse, and is composed primarily of commensal rather than pathogenic mircoorganisms [49]. Aagard et al. [49] thus propose that the maternal bloodstream is the route of transport of bacteria from the maternal mouth to the placenta and possibly represents a mechanism of colonization of the fetal gut by commensal species. If this hypothesis is correct, the benefits of transferring commensal bacteria to the gut of the developing embryo might therefore partially offset the selective pressure to oppose transplacental transmission of pathogenic bacteria through reduced placental invasiveness.

How pathogens enter the placenta and fetus is poorly understood. Bacteria seem to spread directly from cell to cell, or they may enter the host cells from the extracellular matrix and subsequently multiply (reviewed in [14]). Several pathogens, including bacteria, protozoa and viruses, capable of infecting the placenta and fetus, have intracellular life stages within maternal cells [14]. For example, bacterial infections can start at the decidua where maternal leukocytes are recruited; thus, infected leukocytes might be a means through which bacteria reach this site [14]. However, some studies on listeriosis in humans and other mammals with invasive hemochorial placentation have found that the syncytiotrophoblast layer (a multinucleated layer at the areas of exchange in hemochorial and endotheliochorial placentae, lacking or being minimal in epitheliochorial placentae; [2]), bathed in maternal blood, can be unexpectedly resistant to listerial infection [50–52]. This resistance seems to be determined by the lack of those cellular receptors in the syncytium that the bacteria normally exploit to enter a host cell in other tissues, and so the syncytium appears to act as a physical barrier to infection [50–52] (but see [53]). If so, the site of origin of infection by pathogens exploiting internalization as a mechanism of transmission should reside elsewhere in the placenta, and the extra-villous trophoblast seems to be the area where most bacterial infections begin. It is still debated, however, which receptors are expressed and where in the placenta, and ultimately how this can lead to infection (for example, evidence suggesting the expression of E-cadherin, exploited by *Listeria*, in the syncytium of the human placenta is controversial; [14,53]).

When further data are available for a large number of host and pathogen species, comparative studies will be particularly valuable to test hypotheses on the coevolution of host defence mechanisms and pathogen infection mechanisms. For example, comparative approaches can help quantify the importance of an intracellular life cycle in successful infection by a diverse range of pathogens. Nonetheless, the current evidence suggests that the presence of possible alternative protective mechanisms in invasive placentation might help explain the relatively weak association we find between invasiveness and bacteria species richness. These mechanisms may include those present in the syncytium (e.g. [50–52]), and the requirement of transferring commensal bacteria to the developing fetus [49].

Counter to predictions, protozoa species richness is greater in mammals with invasive hemochorial placentation. The observation that the syncytium of hemochorial human and mice placentae infected by *Plasmodium*, *Toxoplasma* and *Trypanosoma* is degraded [54,55], suggests that these protozoa can evade additional protective mechanisms, such as lack of receptors for internalization at this placental site, by degrading the syncytium and gaining contact with the underlying mononucleated layer. The mechanisms promoting such degradation are however less clear. *Trypanosoma* appears to secrete proteases that help to degrade collagen in the syncytium and subsequently binds to the receptors of the mononucleate layer to become internalized and transferred to the fetal side of the placenta [54]. As for bacteria, protozoan infections (e.g. toxoplasmosis) can also begin from the decidua and the extravillous trophoblast [55].

We propose that the need to pass maternal antibodies to the offspring prior to birth when postnatal risk of protozoan infection is high might explain our result that hemochorial placentation is associated with high protozoa species richness, and represents an evolutionary selective pressure for retaining invasive placentation. Maternal antibodies can increase neonatal survival and growth rates, and influence the development of the offspring’s immune system [56]. Maternal antibodies, particularly immunoglobulin-G (IgG), pass readily from maternal blood to fetal tissues through an hemochorial placenta, and the concentration of maternal antibodies in the fetus may reach or exceed levels in the maternal blood [2,57,58]. Conversely, fetal concentration of maternal antibodies in species with endotheliochorial placentae is approximately 10% of that in the mother, and transplacental transmission of maternal antibodies is virtually absent in species with non-invasive epitheliochorial placentation [2,57,58].

The yolk sac placenta is also responsible for IgG transfer to the fetus [2,59]. Interestingly this additional extra-embryonic structure is highly developed and functional in rabbits (*Oryctolagus cuniculus)* and rodents, all mammals with hemochorial placentation, but is reduced and non-functional in species with non-invasive placentae, such as artiodactyls [2,57]. Thus, species with hemochorial placentation have two routes for the transfer of maternal antibodies during gestation. Conversely, in species with less invasive placentae neonates are born immunologically incompetent or with low levels of passive immunity, and transfer of maternal antibodies occurs after birth via colostrum [2,57,58,60]. We thus suggest that hemochorial placentation ensures that the newborn is immunologically protected and invasive placentation can be evolutionarily advantageous when neonatal risk of infection is high.

Finally we find no association between placental invasion and virus richness. Why this is the case is unclear but our results indicate that viral transmission may be unaffected by placental morphology.

To conclude, we find mixed support for the hypothesis that non-invasive placentation provides a barrier to transplacental transmission of microparasites. Conversely, our result that invasive placentation is associated with greater protozoa richness–and independent evidence that antibodies transfer is high in hemochorial placentae–suggest a new hypothesis that invasive placentation represents an evolutionary adaptation to increase neonatal protection when postnatal risk of infection is high. To fully understand how placental morphology interacts with parasite pressure, future studies could examine the prevalence of infection pre- and postnatally across species with different placental morphology, and investigate how transmission mechanisms are affected by diversity in placental invasiveness. Based on our findings, we expect that higher prevalence of bacterial infection during gestation is more likely to favor the evolution of non-invasive placentation, while greater postnatal risk of protozoan infection promotes the evolution of invasive placentation. In addition, because host behavioral and ecological traits are known to influence levels of parasitism [33,35], they may also play a key role in the evolution of placental morphology.

## Supporting Information

S1 Data Data table used for the analysis. (XLSX)

S1 File Additional information on the data and analyses. (DOCX)
