# Supplementary material for: Microparasites and Placental Invasiveness in Eutherian Mammals
Source: PLoS One. 2015 Jul 13;10(7):e0132563. doi: 10.1371/journal.pone.0132563 (PMC4500545; doi:10.1371/journal.pone.0132563)
Supplement: S1 File — (DOCX) [file pone.0132563.s002.docx]

Supporting information

S1 File. Additional information on the data and analyses

Appendix A. References for placental morphology

1. Mossman H (1987) Vertebrate fetal membranes: comparative ontogeny and morphology, evolution, phylogenetic significance, basic functions, research opportunities. New Brunswick, NJ: Rutgers University Press.

2. Benirschke K, editor. Comparative placentation. Available: http://placentation.ucsd.edu/index.html. Accessed 22 February 2012.

3. Hamlett W (1932) Observations on the embryology of the badger. Anat Rec 53: 283–303.

4. Elliot MG, Crespi B (2009) Phylogenetic evidence for early hemochorial placentation in eutheria. Placenta 30: 949–967.

5. Goodrowe K, Smak B, Presley N, Monfort S (1996) Reproductive, behavioral, and endocrine characteristics of the Dall’s sheep. Zoo Biol 15: 45–54.

6. Gruenwald P (1973) Lobular structure of hemochorial primate placentas, and its relation to maternal vessels. Am J Anat 136: 133–151.

7. Wislocki G (1926) Remarks on the placentation of a platyrrhine monkey (*Ateles geoffroyi*). Am J Anatomy 36: 467–487.

8. Benirschke K, Miller CJ (1982) Anatomical and functional differences in the placenta of primates. Biol Reprod 26: 29–53.

9. Houston M, Hendrickx A (1975) Placental structure of the sooty mangabey (*Cercocebus atys*). Anat Rec 181: 379–380.

10. Noback CR (1946) Placentation and angiogenesis in the amnion of a baboon (*Papio papio*). Anat Rec 94: 553–567.

11. Burton GJ (1980) Early placentation in the dusky leaf monkey (*Presbytis obscura*). Placenta 1: 187–195. doi:10.1016/S0143-4004(80)80001-4.

12. Njogu A, Owiti GO, Persson E, Oduor-Okelo D (2006) Ultrastructure of the chorioallantoic placenta and chorionic vesicles of the lesser bush baby (*Galago senegalensis*). Placenta 27: 771–779. doi:10.1016/j.placenta.2005.07.003.

Appendix B. Statistical analysis: additional information and tables

Count data are normally analyzed using transformation such as square-root in place of log-transformation [1]. We attempted to use transformations commonly recommended for counts but these produced model residuals that violate some assumptions of the analysis, contrary to log-transformation presented here (see Methods). Results of analyses of placentation and microparasites using other transformations, however, did not differ qualitatively from those presented here. In the analysis of bacteria species richness the likelihood surface for the ML estimation of λ was flat between approximately 0.0 and 0.4. Therefore we repeated the analysis fixing λ at its estimated 95% confidence interval values to assess the influence of λ on the results. Results were qualitatively similar to those in which ML λ value was estimated.

References

1. Quinn, G. & Keough, M. 2002 Experimental design and data analysis for biologists. Cambridge: Cambridge University Press.

**Table A. Microparasite species richness and placentation without controlling for the species richness of other microparasites (models include *Galagoides demidoff*).**

| **Microparasite** | **Bacteria** | | **Protozoa** | | **Virus** |  |
| --- | --- | --- | --- | --- | --- | --- |
| Predictors | t_132_ | p | t_132_ | p | t_133_ | p |
| Citation count | -2.3 | 0.022 | -1.8 | 0.078 | 7.5 | <0.001 |
| (Citation count)^2^ | 3.8 | <0.001 | 3.3 | 0.001 | - | - |
| Endotheliochorial | -2.4 | 0.020 | -0.6 | 0.525 | 0.4 | 0.706 |
| Hemochorial | -5.3 | <0.001 | 6.2 | <0.001 | -0.1 | 0.991 |
| ***Post-hoc* testing** |  |  |  |  |  |  |
| Epitheliochorial | 5.2 | <0.001 | -6.2 | <0.001 | 0.1 | 0.991 |
| Endotheliochorial | 1.8 | 0.069 | -5.5 | <0.001 | 0.3 | 0.740 |
| **Model summary** |  |  |  |  |  |  |
| Lh | -35.5 |  | -10.3 |  | -49.8 |  |
| ML λ | 0.0 |  | 0.0 |  | 0.31 |  |
| R^2^ | 0.40 |  | 0.48 |  | 0.30 |  |

Placentation is coded with dummy variables. Epitheliochorial placentation is used as the reference level; for *post-hoc* testing hemochorial placentation is set as the reference level. For each predictor in the model we report the t-value with degrees of freedom (t_df_) and p-value; for each model we report the model log-likelihood (Lh), the estimated value of the phylogenetic signal in the model residuals as quantified by λ (ML λ), and the amount of variance in microparasite species richness explained by the model (R^2^).

**Table B. Full models of bacteria species richness and placentation with *Galagoides demidoff*, while controlling for other microparasites’ species richness.**

|  | **Bacteria** | |
| --- | --- | --- |
| Predictors | t_130_ | p |
| Citation count | -2.4 | 0.017 |
| (Citation count)^2^ | 2.9 | 0.005 |
| Virus | 6.1 | <0.001 |
| Protozoa | 2.7 | 0.008 |
| Endotheliochorial | -1.3 | 0.197 |
| Hemochorial | -2.3 | 0.026 |
| ***Post-hoc* testing** |  |  |
| Epitheliochorial | 2.3 | 0.027 |
| Endotheliochorial | 0.9 | 0.394 |
| **Model summary** |  |  |
| Lh | -9.1 |  |
| ML λ | 0.31 |  |
| R^2^ | 0.56 |  |

Placentation is coded with dummy variables. Epitheliochorial placentation is used as the reference level; for *post-hoc* testing hemochorial placentation is set as the reference level. For each predictor in the model we report the t-value with degrees of freedom (t_df_) and p-value; for the model we report the model log-likelihood (Lh), the estimated value of the phylogenetic signal in the model residuals as quantified by λ (ML λ), and the amount of variance in bacteria species richness explained by the model (R^2^).
